# The ‘α-synucleinopathy syndicate’: multiple system atrophy and Parkinson’s disease

**DOI:** 10.1007/s00702-023-02653-2

**Published:** 2023-05-25

**Authors:** Jeswinder Sian-Hulsmann, Peter Riederer

**Affiliations:** 1 Department of Human Anatomy and Medical Physiology, University of Nairobi, Nairobi, Kenya; 2 Department of Psychiatry, Psychosomatics and Psychotherapy, Center of Mental Health, University Hospital Würzburg, Würzburg, Germany; 3 Department of Psychiatry, University of Southern Denmark Odense, J.B. Winslows Vey 18, 5000 Odense, Denmark

**Keywords:** Multiple system atrophy, Glial cytoplasmic inclusions, Parkinson’s disease, Lewy bodies, Pathomechanisms, α-synuclein, Neurodegeneration

## Abstract

Multiple System Atrophy (MSA) and Parkinson’s diseases (PD) are elite members of the α-synucleinopathy organization. Aberrant accumulations of the protein α-synuclein characterize them. A plethora of evidence indicates the involvement of these rogue inclusions in a cascade of events that disturb cellular homeostasis resulting in neuronal dysfunction. These two neurodegenerative diseases share many features both clinically and pathologically. Cytotoxic processes commonly induced by reactive free radical species have been associated with oxidative stress and neuroinflammation, frequently reported in both diseases. However, it appears they have characteristic and distinct α-synuclein inclusions. It is glial cytoplasmic inclusions in the case of MSA while Lewy bodies manifest in PD. This is probably related to the etiology of the illness. At present, precise mechanism(s) underlying the characteristic configuration of neurodegeneration are unclear. Furthermore, the “prion-like” transmission from cell to cell prompts the suggestion that perhaps these α-synucleinopathies are prion-like diseases. The possibility of some underlying genetic foul play remains controversial. But as major culprits of pathological processes or even single triggers of PD and MSA are the same—like oxidative stress, iron-induced pathology, mitochondriopathy, loss of respiratory activity, loss of proteasomal function, microglial activation, neuroinflammation—it is not farfetched to assume that in sporadic PD and also in MSA a variety of combinations of susceptibility genes contribute to the regional specificity of pathological onset. These players of pathology, as mentioned above, in a synergistic combination, are responsible for driving the progression of PD, MSA and other neurodegenerative disorders. Elucidating the triggers and progression factors is vital for advocating disease modification or halting its progression in both, MSA and PD.

## Introduction

Multiple System Atrophy (MSA) is a neurodegenerative disorder characterized by marked motor impairment, parkinsonian features, cerebellar ataxia, tremor, cortico-spinal tract dysfunction, autonomic abnormalities, and deficits in executing functions (Krismer and Wenning 2017; Jellinger 2020; Kübler et al. 2023). It chiefly comprises of two major variants depending on the dominant clinical phenotype, one of which is MSA-P. It also demonstrates parkinsonian features coupled with striatonigral degeneration. In contrast, the other variant exhibits cerebellar ataxia (MSA-C) and olivopontocerebellar atrophy. The motor deficits exhibited by the two variants result from the degeneration (MSA-P) and atrophy (MSA-C) observed in these regions. MSA-C has a higher incidence (67–84%) in the Asian population (Ozawa and Onodera 2017), whereas MSA-P is common in western countries (70–80%) (Jellinger 2019; Wenning et al. 2013). Unfortunately, there is no effective treatment for halting/slowing the process of neuronal degeneration. l-Dopa-therapy, although of substantial benefit in PD, shows a poor response in MSA.

MSA shares many clinical and neuropathological characteristics with Parkinson’s disease, another α-synucleinopathy (PD; Wenning et al. 2013). The degenerative processes occurring in MSA and PD (Riederer et al. 2019; Sian-Hulsmann and Riederer 2021) appear multifactorial.

Although the vicious primary trigger that initiates the pathological processes is unclear, findings from MSA human post-mortem studies and animal models have reflected the involvement of several factors that may exert an instrumental role in the pathogenesis. These include microglial activation and neuroinflammation (Vieira et al. 2015), oxidative stress (Jellinger and Wenning 2016), reduced respiratory chain activity (Foti et al. 2019), and impaired mitochondrial function (Nakamoto et al. 2018), proteasomal-autophagy degradation dysfunction (Monzio Compagnoni et al. 2018), extensive demyelination (Matsuo et al. 1998) and aberrant accumulation of α-synuclein (Spillantini et al. 1998). Many of these changes are also reported in PD (Riederer et al. 2019). The biochemical and pathophysiological alterations probably play a crucial role in executing cytotoxic processes. However, whether these are of primary or secondary importance for the pathology of PD and MSA is still unknown.

Although the precise causative mechanisms of MSA are obscure, the occurrence of misfolded α-synuclein protein inclusions in brain areas demonstrating marked degeneration represents a characteristic neuropathological feature of the disease. It endorses the involvement of this rogue protein in a neuronal massacre. Similar to PD, it is a member of the elite α-synucleinopathies organization. Other neurodegenerative diseases in this group include, e.g., dementia with Lewy bodies (DLB), Parkinson’s disease dementia (PDD), and Progressive Supranuclear Palsy (PSP) (Dickson et al. 2007; Jellinger 2011, 2017). α-Synucleinopathies share a common feature of α-synuclein aggregate pathology, although this inclusion may differ molecularly in the various syndromes. It has been suggested that post-translational modifications ascribe to an abnormal protein assembly and the consequent α-synuclein aggregates (Manzanza et al. 2021). The misfolding, oligomerisation, and aggregation of α-synuclein is perhaps the most common pathophysiology of these syndromes (Trojanowski and Lee 2006). Despite this, there are different pathologic α-synuclein inclusions and clinical manifestations. This begs the question; what factor(s) determines the disease that finally manifests? Is it the different α-synuclein strain present? Why are only specific brain areas susceptible to the onslaught of the disease?

The accumulation of α-synuclein may be reflective of some malfunction in the breakdown of the protein by the autophagy-lysosomal system, which may be directedly related to the pathogenesis of the disease (Xilouri et al. 2016). Alternatively, an underlying genetic component may prescribe the production of α-synuclein mutations resulting in its abhorrent amassing. Interestingly, an association between α-synuclein gene polymorphism and the genes related to oxidative stress and inflammation appears to increase susceptibility to MSA. However, other studies have not reported this (Jellinger 2018a, b). Also, MSA is a sporadic disease without any major genetic association with its pathogenesis (Jellinger 2018a, b). Indeed, familiar MSA is uncommon (Fujioka et al. 2014), and no gene mutation characterizing a familiar form has been identified. Nevertheless, some genetic components in the pathogenesis warrant further research since some studies have indicated a possible involvement of genetic and epigenetic factors (Vanacore et al. 2001; Sturm and Stefanova 2014). Epidemiological studies have even suggested a possible association of MSA to epigenetic factors and environmental neurotoxin (Hanna et al. 1999; Sturm and Stefanova 2014), despite modest sample numbers and the inability to repeat them, cast a shadow of doubt over these conclusions.

Recently, using single-cell RNA sequencing, Kamath et al. (2022) identified 10 transcriptionally distinct populations of dopaminergic neurons in the substantia nigra pars compacta (SNpc). They reported that in the case of PD patients, one out of the 10 dopaminergic-producing cell populations marked by the gene AGTR1 was present in the ventral tier of the SNpc. This area suffers from the onslaught of degenerative processes (Kamath et al. 2022). Thus, this provides persuasive evidence for the involvement of genetic components in determining the vulnerability of neurons in PD. The diversity in a hereditary component may distinguish pathogenesis exhibited by different α-synucleinopathies despite sharing some degenerative processes and clinical manifestations.

In PD, the α-synuclein aggregates appear as eosinophilic inclusions referred to as Lewy bodies (LB) present in the residual dopaminergic neurons in the area exhibiting marked cell loss in the substantia nigra pars compacta. They probably account for motor dysfunction (Gibb and Lees 1988). Subsequently, as the disease progresses, LB pathology, in a prion-like manner, spreads to other brain regions, accounting for some of the clinical symptoms of the disorder. In contrast, the filamentous α-synuclein accumulates in MSA and shows widespread neuronal and axonal multisystem degeneration (Jellinger 2018a). It occurs in the oligodendroglia, referred to as glial cytoplasmic inclusions (GCI) (Trojanowski and Revesz 2007). α-Synuclein positive GCI is the pathological hallmark of MSA. There is a selective neuronal loss and axonal degeneration with a focus on striatonigral and OPC systems suggesting a transsynaptic striatonigral degeneration (Jellinger 2019). In addition, MSA degenerative processes also afflict neurons in the subthalamic nucleus and globus pallidus (Jellinger 2014). Myelin disturbances and microglial activation align with neuroinflammation findings (Hoffmann et al. 2019). A comparison of α-synuclein content in GCI and LB shows 11.9% in GCI and 8.5% in LB (McCormack et al. 2016). LB has been demonstrated in 10.7–22.7% of MSA cases (Koga et al. 2017; Jellinger 2019). Of note, there is a difference in the phosphorylation pattern of α-synuclein between PD and MSA (Yamasaki et al. 2019). In addition, oligodendroglial changes are more widespread than α-synuclein-positive GCI and are regarded as the main drivers of pathology (Jellinger 2019).

Furthermore, α-synucleinopathy exhibited in MSA (GCI), PD (LB), and diffuse LB disease (DLB) differs in structural and chemical characteristics (Jellinger 2021; Ayers et al. 2022). Additionally, the native α-synuclein protein has acidic glutamate at residue 46 while in familial PD, it is a mutated basic lysine (Ayers et al. 2022). Nevertheless, the appearance of these inclusions in an almost congruent pattern to neuronal death suggests its pivotal involvement in the destructive neuronal pathways.

Also, this positive correlation between the GCI population and the severity of neuronal degeneration supports the notion that MSA is fundamentally and prototypically an oligodendrogliopathy (Wenning et al. 2008) that evokes neuronal destruction. Additionally, the pathogenic effects of GCI and other α-synuclein inclusions have been demonstrated by experiments using transgenic animals overexpressing α-synuclein, particularly in the oligodendrocytes (Yazawa et al. 2005; Stefanova and Wenning 2015). Equally, oligodendrocytes have also been implicated in the pathogenesis of PD (Dean et al. 2016). MSA is considered to be primarily a glial disease. Perhaps these aberrant α-synuclein accumulations exert their cytotoxic effect via altering the neuronal homeostasis resulting in neuronal destruction and myelin autophagocytosis. Indeed, impaired chaperone-mediated autophagy (substrate proteolysis) in rats has deleterious effects on the neurons (Brekk et al. 2019).

Myelin dysregulation is primary to the production of glial inclusion, as reported in early MSA and demonstrated by changes in the myelin protein and p25α before GCI (Wenning et al. 2008). Thus suggesting “sick” oligodendrocytes may result in myelin dysfunction since one of their primary functions is myelin production (Ettle et al. 2016). This may, in turn, interfere with the physiological functioning of neurons since oligodendrocytes and myelin play a key role in preserving neuronal circuits and communication. Consequently, this may augment the vulnerability of neurons to cytopathogenic processes (such as oxidative stress and neuroinflammation), leading to the production of these inclusions in MSA. Similarly, myelin dysfunction has also been suggested in PD, whereby polymorphism (rs616147) of the myelin-associated oligodendrocyte basic protein has also been documented as a risk factor for the disease (Siokas et al. 2021).

## The role of oxidative stress

The active participation of oxidative stress in the torrent of neurodegenerative processes in MSA and PD is well established. It has been postulated that reactive oxygen species are elevated in such a state, which may change intracellular communication. This, in turn, dysregulates the neuroinflammatory system and microglia activation. Oxidative stress plays a crucial role in immune-induced cell death. More importantly, the excessive production of cytotoxic free radicals may overwhelm the cellular antioxidant defenses, thereby exposing the cells to the assault of pathogenic reactions and resulting in the onset of the neurodegenerative disorder (Mendiola et al. 2020). Many studies have suggested the involvement of iron, mitochondrion, endoplasmic reticulum or accumulation of α-synuclein in cellular oxidative stress (Sian-Hulsmann and Riederer 2020; Riederer et al. 2019 for reviews).

Iron has been shown to cause oligomerization of α-synuclein. This is associated with free radical production, which would collectively exacerbate the degree of oxidative stress-induced cell death. Additionally, α-synuclein aggregates interact with lipid cell membranes to induce ferroptosis-facilitated cell destruction (Angelova et al. 2020).

Iron-induced oxidative stress in MSA has been highlighted recently by Kaindlstorfer et al. (2018). As in PD, iron may directly act on α-synuclein and may provoke an insoluble molecular structure (Riederer et al. 2019; Lee and Lee 2019), which aggregates as glial cytoplasmic inclusions (Probst-Cousin et al. 1998), or iron induces oxidative stress mechanisms which may contribute to disturbances of glial function and finally neuronal death. Since α-synuclein is a ferrireductase, this protein expression depends on iron-related translational processes (Sian-Hulsmann and Riederer 2020). Dexter et al. (1991) described increased iron concentration in MSA, especially in the putamen. Furthermore, oligodendrocytes, which contain high amounts of iron, are assumed to contribute to iron-induced oxidative stress after iron release from ferritin, thereby causing glial cytoplasmic inclusions (Kaindlstorfer et al. 2018). Of note, is the reduction of the cellular antioxidant GSH (reduced glutathione) in the substantia nigra pars compacta in PD (Sian-Hulsmann and Riederer 2021), in contrast with the increase of GSH in MSA (196%) coupled with a reduction of GSSG (60%) in the globus pallidus (Sian et al. 1994). However, in the study by Fitzmaurice et al. (2003), nigral GSH (−20%) did not reach significance in MSA and GSH levels were normal in all other extra-nigral brain areas in MSA and Progressive Supranuclear Palsy (PSP). This assumes that astrocyte-related GSH synthesis and neuronal GSH synthesis (Dringen et al. 1999) are hardly affected in MSA, which may be important to differentiate MSA and PD.

Under physiological conditions the reactive oxygen species/free radicals generated from the mitochondrial electron transport chain are inactivated by the anti-oxidants such as GSH and superoxide dismutase. However, in the disease state these cellular defense mechanisms may be overwhelmed by the excessive production of free radicals resulting in oxidative stress and cytotoxic events. Indeed, the mitochondria has been pointed to be the key site for the generation of free radicals in the hyperactive neurons (Helwig et al. 2022). Foti and colleagues (2019) measured respiratory chain activities in cerebellar and occipital white matter and showed decreased complex II/III activity in the mitochondrial electron transport chain, whereas there was an elevation of complex I and IV activity in MSA cerebellar white matter. This finding is at variance with nigral mitochondrial pathology in PD (Riederer et al. 2021). Another difference related to the pathology of MSA and PD seems to be the association with the white matter in the cerebellum and occipital lobe, which are affected in MSA but show minimum pathology of Lewy neurites in PD (Foti et al. 2019).

Whereas, under pathological conditions there may be some malfunction in the endoplasmic reticulum, thereby disrupting the regulation of calcium in the cytosol. Consequently, this may prompt an increase in the uptake of these ions by the mitochondrial calcium uniporter and this can trigger the production of free radicals from the activated mitochondrial electron transport chain. In addition, the excess cellular calcium ions may activate mitochondrial-associated apoptotic pathways (Hoozemans et al. 2007).

Endoplasmic stress may also be prompted by the misfolded α-synuclein inclusions. By this, it may trigger by binding to the chaperones of the endoplasmic reticulum and producing a disorder in vesicle trafficking from the endoplasmic reticulum to the Golgi body (Hammadi et al. 2013) (Tables 1, 2).

**Table 1:** Similarities of pathological processes in Parkinson’s disease and Multiple System Atrophy

Accumulation of α-synuclein
Impaired mitochondrial function
Reduced respiratory chain activity
Malfunction of the endoplasmic reticulum
Iron-induced oxidative stress /ferroptosis and iron-related translational processes
Autophagy/lysosomal dysfunction
Proteasomal dysfunction
Microglial activation
Neuroinflammation
Multifactorial

For references see text

**Table 2:** Dissimilarities of pathological processes in Parkinson’s disease and Multiple System Atrophy

α-Synuclein aggregates spread from neuron to neuron in acute mouse experiments but not to oligodendrocytes, but do so in long-term studies
Uncertainty, whether α-synuclein pathology in oligodendrocytes is sufficient to reproduce MSA phenotype
Familiar cases have been identified in PD but are uncommon in MSA
α-synucleinopathies in PD and MSA differ in structural and chemical characteristics
Phosphorylation pattern of α-synuclein is different between PD and MSA
Oligodendroglial changes are more widespread than α-synuclein-positive GCIs in MSA
LBs are characteristic in the SNpc but rare in oligodendrocytes of PD, while in MSA α-synuclein-positive glial cytoplasmic inclusions are characteristic
Neuronal loss and axonal degeneration are at variance in PD and MSA
In MSA degenerative processes have been identified in the subthalamic nucleus and the globus pallidus
There is a nigro-striatal degeneration in PD, but a striatonigral degeneration in MSA; L-DOPA is beneficial in PD but has a poor response in MSA
There is an increase of iron in the SN in PD but an increase of iron in the putamen in MSA
The pathology of the mitochondrial electron transport chain is at variance in PD and MSA
There is a decrease of GSH in the SN of PD, but eventually an increase of GSH in the globus pallidus of MSA
Myelin dysfunction is mild in PD but extensive in MSA
White matter pathology is at variance in PD and MSA

For references see text

## Onset of α-synucleinopathies

α-Synuclein research has developed since the seminal publication of Braak et al. (2003). Based on α-synuclein pathology only, Braak et al. (2003) have created a “staging protocol”. Braak et al. proposed that the pathology of PD starts in the enteric nervous system (and possibly also in the olfactory system). However, the gut–brain-axis concept holds for only a part of patients with PD and has been discussed in detail and summarized by Kalaitzakis et al. (2008), Parkkinen et al. (2008) and Jellinger (2018a, 2019). The conclusion is that, in addition, there must be other ways of pathological α-synuclein spreading. Therefore, the gut–brain-axis hypothesis has been modified and enlarged to the “bottom-up–top–down” hypothesis (Urban et al. 2020 for review) or, more recently, to the “body-first”—(Engelender and Isacson 2017; Foffani and Obeso 2018) “brain-first” model of Lewy body (LB) diseases (Borghammer et al. 2021). Based on experimental work and human post-mortem analyses, these new concepts share the idea that PD pathology may also start in brain areas and progress to peripheral organs, including the enteric nervous system. In addition to the vagal pathology, the peripheral autonomic nervous system is involved in spreading α-synuclein pathology (reviewed by Borghammer et al. 2021, see also Isonaka et al. 2022).

Body-first and brain-first subtypes, for example, show distinct clinical and (neuro) pathological findings with regard to symmetry–asymmetry of dopaminergic denervation resp. motor symptoms at diagnosis, progression rate/motor symptom progression, hyposmia, REM-sleep behavior, locus coeruleus degeneration, sympathetic denervation, autonomic symptoms and parasympathetic denervation (Borghammer et al. 2021).

The initiation and progression of α-synuclein in PD have been reviewed recently by Tofaris (2022). Aberrant interactions of α-synuclein and lipids or evasion of proteostatic defense at the site of synaptic terminals may be responsible for α-synuclein aggregation (Tofaris 2022). As described above in detail, multiple forms of interactions and pathological outcomes may potentially lead to various subtypes of PD. Indeed, Tofaris (2022) hypothesizes that there may be variants of a dominant α-synuclein strain, which occur across brain areas and/or patients. But if this is true, “a single immunotherapy or anti-aggregation therapy will never be efficacious across the whole disease” (Tofaris 2022).

Lipid-mediated stabilization of neuroprotective α-synucleinH (α-SH), the cytosolic helically folded, multimeric form of α-synuclein, resists disease-associated changes. In contrast, the cytosolic unfolded, monomeric form of α-SU is aggregation-prone and can misfold into soluble, toxic oligomers, protofilaments, and amyloid fibrils forms associated with pathology (de Boni et al. 2022). The likelihood of fibril formation and LB inclusion increases when the equilibrium of α-SH/α-SU shifts towards the aggregation-prone α-SU in DLB and PD patients (de Boni et al. 2022). Moreover, single molecule techniques have revealed that different oligomers may assemble during the formation of α-synuclein fibrils, but only those which are proteinase K resistant, i.e. containing ß-sheet conformation, damage cells (Tofaris 2022).

Posttranslational modifications (PTM) of α-synuclein play a major role in its pathology. Phosphorylated, nitrated, and oxidized forms seem to exist. Sonustun et al. (2022) examined pS87, pS129 and nY39. Quantification of the LB scores revealed that pS129 α-synuclein was the dominant and earliest α-synuclein PTM, followed by nY39 α-synuclein. In contrast, lower amounts of pS87 α-synuclein appeared later in the disease progression of PD (Sonustun et al. 2022). A higher expression of pS87 has been detected in AD, MSA, and DLB (Paleologou et al. 2010). PTMs of α-synuclein display variable abundance at different sites within LBs, Lewy neurites, and glial cytoplasmic inclusion. However, it seems unclear which α-synuclein PTMs appear within aggregates throughout disease pathology (Sonustun et al. 2022). Markers of oxidized proteins, lipids, and DNA are upregulated in dopaminergic neurons of PD (Dias et al. 2013).

In this respect, the role of iron-induced oxidative stress on tyrosine residues 39, 122, 129, and 133 of α-synuclein has been reviewed in detail by Riederer et al. (2019). Of note, stereotactic injection of α-synuclein preformed fibrils into the striatum of mouse brains after neonatal brain iron enrichment caused intrastriatal microglia accumulation, which was alleviated by iron in a dose-dependent way (Dauer Nee Joppe et al. 2021). Of interest, Jin et al. (2022) demonstrated that oxidation of tyrosine produces DOPAnization and leads to the formation of oligomers.

Experimental work shows that microglia exposed to α-synuclein establish a cellular network through the formation of F-actin-dependent intercellular connections, which transfer α-synuclein from overloaded microglia to neighboring naive microglia where the α-synuclein cargo gets rapidly and effectively degraded, thus improving pathogenic α-synuclein clearance (Scheiblich et al. 2021).

## Progression of α-synuclein pathology

It has been reported that in PD α-synuclein inclusions are spread extensively in the central nervous system, enteric nervous system, submandibular gland, adrenal medulla, and sympathetic ganglia (Wakabayashi 2020). α-synuclein aggregates have been suggested to spread in a prion-like fashion from cell to cell and contribute to the progression of the disease (Woerman et al. 2018). The presence of α-synuclein pathology supports this notion in early PD only in areas governing motor function. However, it proliferates to other regions in the brain as the illness progresses (Henderson et al. 2019; Du et al. 2020; Guo et al. 2022). Interestingly, α-synuclein pathology and LB are exhibited in some of the fetal grafted dopamine neurons (11–12%) in PD (Li et al. 2016), thereby reflecting the spread of α-synuclein pathology from the host to grafted neurons. Additionally, administering human α-synuclein fibrils into the striatum of macaque monkeys evoked PD-like neuropathology, including LB and bulky α-synuclein-positive intracytoplasmic inclusions (Kawakami et al. 2021). Furthermore, this study (Kawakami et al. 2021) supported the prion-like spread of the α-synuclein pathology through axonal-synapses linkage. The transmission hypothesis of pathological α-synuclein suggests that the initial seed of abnormal α-synuclein may be released from the “afflicted” neuron, which may be accepted by a neighboring unaffected cell and thus trigger the misfolding of the protein there (Henderson et al. 2019).

The notion is of importance that spreading of α-synuclein aggregates from neuron to neuron in mouse experiments has been demonstrated while spreading to oligodendrocytes was not observed (Watts et al. 2013). However, long-term studies show that WT mice injected with mouse α-synuclein preformed fibrils develop neuronal α-synuclein pathology after short time, while oligodendroglial α-synuclein emerges after longer time post injection (Uemura et al. 2019) thus being evidence for a neuron to oligodendroglia transmission of α-synuclein pathology.

Findings using a novel gut–brain α-synuclein transmission mouse model, suggest a transneuronal mode of propagation of the α-synuclein inclusions, and it supports Braak’s hypothesis for gut–brain spread via the vagus nerve in PD (Kim et al 2019). The dorsal motor nucleus of the vagus has been indicated to be the main area of deposition of α-synuclein aggregates and more importantly, it appears to execute an important part in the proliferation of this rogue protein in the CNS and in other areas of the body (Musgrove et al. 2019).

Interestingly, in vitro experiments demonstrate a positive association between oxidative stress and the distribution of α-synuclein pathology. The findings from a study using the α-synuclein mouse model suggest, that neuronal hyperactivity appears to cause cellular havoc by supporting and enhancing oxidative and nitrative stress, and aggregation of the nitrated form of α-synuclein (Helwig et al 2022). Therefore, oxidative stress does contribute to the spreading of the misfolded α-synuclein aggregates. Additionally, the nitrated α-synuclein is the most transferable form (Musgrove et al. 2019) and neurotoxic (Yu et al. 2010). The toxicity related to the nitrated α-synuclein is more likely to be associated with its modulation of the inducible nitric oxide synthase (iNOS)/FAK processes rather than the production of cytotoxic oligomers (Liu et al. 2011). The iNOS/FAK alliance of pathways exerts a pivotal role in toll receptor-linked macrophage mobilization (Maa et al. 2011).

Even the mode and conditions required to spread α-synuclein pathology differ between the syndromes. Interestingly, it has been suggested that specific requirements are warranted for the release of PD α-synuclein harnessed within LB (Ayers et al. 2022). In transgenic mice (Tg SNCA *A53), the transmission of MSA α-synuclein strains was reported (Watts et al. 2013), whereas no transfer was found using α-synuclein strains from PD or DLB brain homogenates. Watts and co-workers (2013) suggested that the pathogenesis of MSA was associated with an aggregation of cytotoxic α-synuclein and that perhaps it was a prion disease due to the similarities of α-synuclein and prions and their mode of transmission. A striking and fundamentally comparable feature of α-synuclein is its prion-like predilection for accumulating and transforming into a pathogenic protein (Jellinger 2021; Woerman et al. 2018). However, others have disputed this since the α-synuclein aggregates in the oligodendrocytes of transgenic mice were not like the characteristic GCI observed in MSA (Yazawa et al. 2005). In addition, overexpression of α-synuclein in oligodendrocytes did not induce neuroinflammation, showed modest neural/oligodendroglial degeneration and moderate motor impairment accompanied by filamentous inclusions that were not shown to be identical to GCI (Yazawa et al. 2005). Furthermore, PLP-promotor driven expression of α-synuclein induced microglial activation but only a mild motor phenotype (Stefanova and Wenning 2015). Therefore, overexpression of α-synuclein in oligodendrocytes may not be sufficient to reproduce the human MSA phenotype. However, transgenic mouse lines expressing human α-synuclein under the control of the murine myelin basic protein promotor showed that those mice expressing high levels of hα-synuclein displayed severe neurological alterations, accumulated hα-synuclein-immunoreactive inclusions in oligodendrocytes along the axonal tracts in the brainstem, basal ganglia, cerebellum and neocortex. The inclusions reacted with antibodies against phospho-serine (129) hα-synuclein and ubiquitin. The oligodendrocytic inclusions were composed of fibrils and accompanied by mitochondrial alterations and disruptions of the myelin lamina in the axons, suggesting that α-synuclein in oligodendrocytes promotes neurodegeneration and mirrors features of MSA (Shults et al. 2005). In addition, mouse models that express α-synuclein specifically in oligodendrocytes through cell-type specific promotors are of interest to elucidate the question, of whether the accumulation of misfolded α-synuclein is causal for MSA (Lee et al. 2019).

Nevertheless, there seems to be a distinction between prions and prion-like (Jellinger 2021), thus suggesting that the prion-like spread of pathology in MSA and PD does not necessarily mean that α-synuclein is a prion. There is an urgent need to design animal/primate experimental models which simulate the formation of the disease-specific α-synuclein inclusions. This would provide the dynamics underlying the pathomechanisms for these α-synucleinopathies and offer specific target therapeutic interventions.

## Other associations with α-synucleinopathies

The high risk of dementia in Lewy body disorders (LBD) is associated with constipation, orthostatic hypotension, and RBD, markers of the body-first subtype of LBD, as reviewed by Borghammer et al. (2021). These authors also suggest that patients with a caudal–rostral distribution of LB pathology are at an elevated risk of dementia in contrast to brain-first PD patients, who are RBD negative and display dopamine degeneration first and show an amygdala-centered distribution of pathology, leading to a slower progression towards dementia (Borghammer et al. 2021). Furthermore, the pathology in synucleinopathies may be further accelerated by the presence of tau or Aß pathology, as in the case of DLB (de Boni et al. 2022; Jellinger 2011, 2022). Moreover, several lines of evidence suggest putative pathogenic modifications to nuclear α-synuclein in DLB (Koss et al. 2022).

As mentioned, α-synuclein pathology is predominantly in dopaminergic systems but affects many other neuronal systems. As such, the notion is of interest that human α-synuclein overexpression in mouse serotoninergic neurons triggers progressive accumulation, phosphorylation, and aggregation of hα-synuclein protein in the serotoninergic system, resulting in a depressive-like phenotype, thus nicely modeling depression and anxiety in PD, which often precede the onset of motor symptoms (Miquel-Rio et al. 2022; Przuntek et al. 2004). As outlined, it is evident that α-synuclein pathology is highly complex. In addition, primary or secondary pathological processes synergistically influence α-synuclein pathology, for instance, other proteinopathies developing parallel to α-synuclein pathology. Moreover, the role of neuromelanin within the pathological process of α-synuclein is not fully understood (Wulf et al. 2022; Nagatsu et al. 2022; Zecca et al. 2004). Finally, individual genetic specialities, including gene assembly and gene connectivities, have to be considered necessary in this respect.

## Future research areas of focus

Finally, it is crucial to investigate and elucidate the following elements to provide a better understanding of the labyrinth of pathomechanisms operating in α-synucleinopathies;

- Is it possible to stop the “conformational change” of native α-synuclein to the rogue version, which has the propensity to misfold and accumulate within neurons/oligodendroglia cells?
- At what disease stage(s) would antioxidant therapy be efficacious? Producing reactive oxygen species that initiate oxidative stress induces harmful neuroinflammation and neurodegeneration. It has been suggested that mobilizing T cells with anti-inflammatory potency can provide neuroprotection by regulating neuroinflammatory processes (Solleiro-Villavicencio and Rivas-Arancibia 2018).
- Would it be possible to “re-set” and regulate the α-synuclein autophagy-lysosomal system? It would be efficacious for putative protective agents to recognize the aberrant α-synuclein aggregates to activate proteostatic defense mechanisms.
- How can cell–cell transmission and thus spread of α-synuclein pathology be immobilized? This may hold the key to halting or impeding disease progression. The α-synuclein transmission must be considered a potential target site for successful disease-modifying therapies.
- Given the significant pathological role of post-translational modifications, perhaps effective treatment must be orchestrated at a genetic level.

Thus, there seems to be a host of promising possibilities for selectively targeting and halting the progression of disease processes. However, the complexities of the pathological mechanisms and the lack of complete understanding of the α-synucleinopathies make it a real challenge.

## Data Availability

The authors produced no data to write this review. The data used is contained in the publications listed in the reference section.
